# New tools to monitor *Pseudomonas aeruginosa* infection and biofilms *in vivo* in *C. elegans*

**DOI:** 10.3389/fcimb.2024.1478881

**Published:** 2024-12-16

**Authors:** Feng Xue, Martina Ragno, Sarah A. Blackburn, Michael Fasseas, Sushmita Maitra, Mingzhi Liang, Subash Rai, Giulia Mastroianni, Frederique Tholozan, Rachel Thompson, Laura Sellars, Rebecca Hall, Chris Saunter, David Weinkove, Marina Ezcurra

**Affiliations:** ^1^ School of Biosciences, University of Kent, Canterbury, United Kingdom; ^2^ Magnitude Biosciences Limited, NETPark Plexus, Sedgefield, United Kingdom; ^3^ Quadram Institute Bioscience, Norwich Research Park, Norwich, United Kingdom; ^4^ The NanoVision Centre, Queen Mary University of London, London, United Kingdom; ^5^ Perfectus Biomed Group, Sci-Tech Daresbury, Chesire, United Kingdom; ^6^ Department of Biosciences, Durham University, Durham, United Kingdom

**Keywords:** *Pseudomonas aeruginosa*, biofilms, quorum sensing, *C. elegans*, antimicrobial resistance

## Abstract

**Introduction:**

Antimicrobial resistance is a growing health problem. Pseudomonas aeruginosa is a pathogen of major concern because of its multidrug resistance and global threat, especially in health-care settings. The pathogenesis and drug resistance of *P. aeruginosa* depends on its ability to form biofilms, making infections chronic and untreatable as the biofilm protects against antibiotics and host immunity. A major barrier to developing new antimicrobials is the lack of *in vivo* biofilm models. Standard microbiological testing is usually performed *in vitro* using planktonic bacteria, without representation of biofilms, reducing translatability. Here we develop tools to study both infection and biofilm formation by *P. aeruginosa in vivo* to accelerate development of strategies targeting infection and pathogenic biofilms.

**Methods:**

Biofilms were quantified in vitro using Crystal Violet staining and fluorescence biofilm assays. For in vivo assays, *C. elegans* were infected with *P. aeruginosa* strains. Pathogenicity was quantified by measuring healthspan, survival and GFP fluorescence. Healthspan assays were performed using the WormGazerTM automated imaging technology.

**Results:**

Using the nematode *Caenorhabditis elegans* and *P. aeruginosa* reporters combined with *in vivo* imaging we show that fluorescent P. aeruginosa reporters that form biofilms *in vitro* can be used to visualize tissue infection. Using automated tracking of *C. elegans* movement, we find that that the timing of this infection corresponds with a decline in health endpoints. In a mutant strain of P. aeruginosa lacking RhlR, a transcription factor that controls quorum sensing and biofilm formation, we find reduced capacity of P. aeruginosa to form biofilms, invade host tissues and negatively impact healthspan and survival.

**Discussion:**

Our findings suggest that RhlR could be a new antimicrobial target to reduce *P. aeruginosa* biofilms and virulence in vivo and *C. elegans* could be used to more effectively screen for new drugs to combat antimicrobial resistance.

## 1 Introduction

The Gram-negative bacterium *Pseudomonas aeruginosa*, a common and opportunistic pathogen, causes disease in a variety of hosts (Rahme et al., 1997; C. et al., 2011; Tan et al., 1999). In humans, *P. aeruginosa* can cause serious complications, form systemic infections in immunodeficient patients and develop into chronic infections (Govan and Deretic, 1996; Lieberman and Lieberman, 2003; Lyczak et al., 2000). *P. aeruginosa* infections are characterized by antibiotic resistance, limited treatment options and high mortality, and outbreaks caused by multidrug resistant strains are on the rise (Fisher et al., 2005; Obritsch et al., 2005; K. et al., 2001). Pathogenesis and drug resistance of *P. aeruginosa* depends on its ability to form biofilms, which make infections chronic and untreatable as the biofilm protects against antibiotics and host immunity (Roy et al., 2018). Although biofilms are central to chronic *P. aeruginosa* infections, research examining *P. aeruginosa* virulence has largely focused on planktonic bacteria or biofilm studies performed *in vitro* or *ex vivo*, reducing translatability and creating a barrier to the development of effective antimicrobials to limit chronic *P. aeruginosa* infections (Highmore et al., 2022).

Biofilm-related infections are challenging to study and monitor *in vivo* in because they are typically internal. While e.g. surface wounds can be monitored in real time, analysis of internal biofilms are typically carried out postmortem on *ex vivo* tissue. There are a few examples of *in situ in vivo* monitoring of biofilms using advanced imaging techniques, such as microcomputed tomography and micropositron emission tomography but these techniques are highly specialized and expensive (Guzmán-Soto et al., 2021). Developing novel *in vivo* approaches that allow high-throughput assays to study biofilm-related infections would increase the understanding of bacterial biofilms and interactions between biofilms and host, and could lead to the development of new strategies targeting antimicrobial resistance and biofilms.

The model organism *Caenorhabditis elegans* offers a valuable tool to study infection that can be developed to perform detailed studies of biofilm formation *in vivo*, increasing the understanding of bacterial physiology within biofilms and interactions between biofilms and the host. *C. elegans* has a small size and rapid generation time and offers an extensive research toolkit including functional assays to monitor health outputs and physiology. *C. elegans* is susceptible to human pathogens and *C. elegans-P. aeruginosa* infection models are particularly useful, as many *P. aeruginosa* virulence-related factors are conserved across widely divergent taxa from nematodes to plants to mammals (Kim and Ausubel, 2005; Rahme et al., 1995; Tan and Ausubel, 2000), and the human innate immune system shares many characteristics with that of *C. elegans*. *C. elegans* feed on bacteria and *C. elegans* are infected with bacterial pathogens by simply transferring worms from their normal laboratory food, the *Escherichia coli* strain OP50, to a lawn of the pathogen of interest growing on agar medium. The ease of *C. elegans* infection makes it an attractive model for high-throughput screening to identify attenuated and hypervirulent strains and as a first stage for testing novel antibiotic compounds. In addition, *C. elegans* is transparent, making it possible to image fluorescent reporters and infection in living animals in real time, while bypassing ethical implications of studying pathogenicity in mammalian *in vivo* models. Thus, *C. elegans* has potential to be developed as a new model to study pathogenic biofilms *in vitro*. Here we report that quorum sensing (QS), a signaling process by which *P. aeruginosa* regulates biofilm formation, is required for *P. aeruginosa* pathogenicity in *C. elegans*.

**Figure 1:**
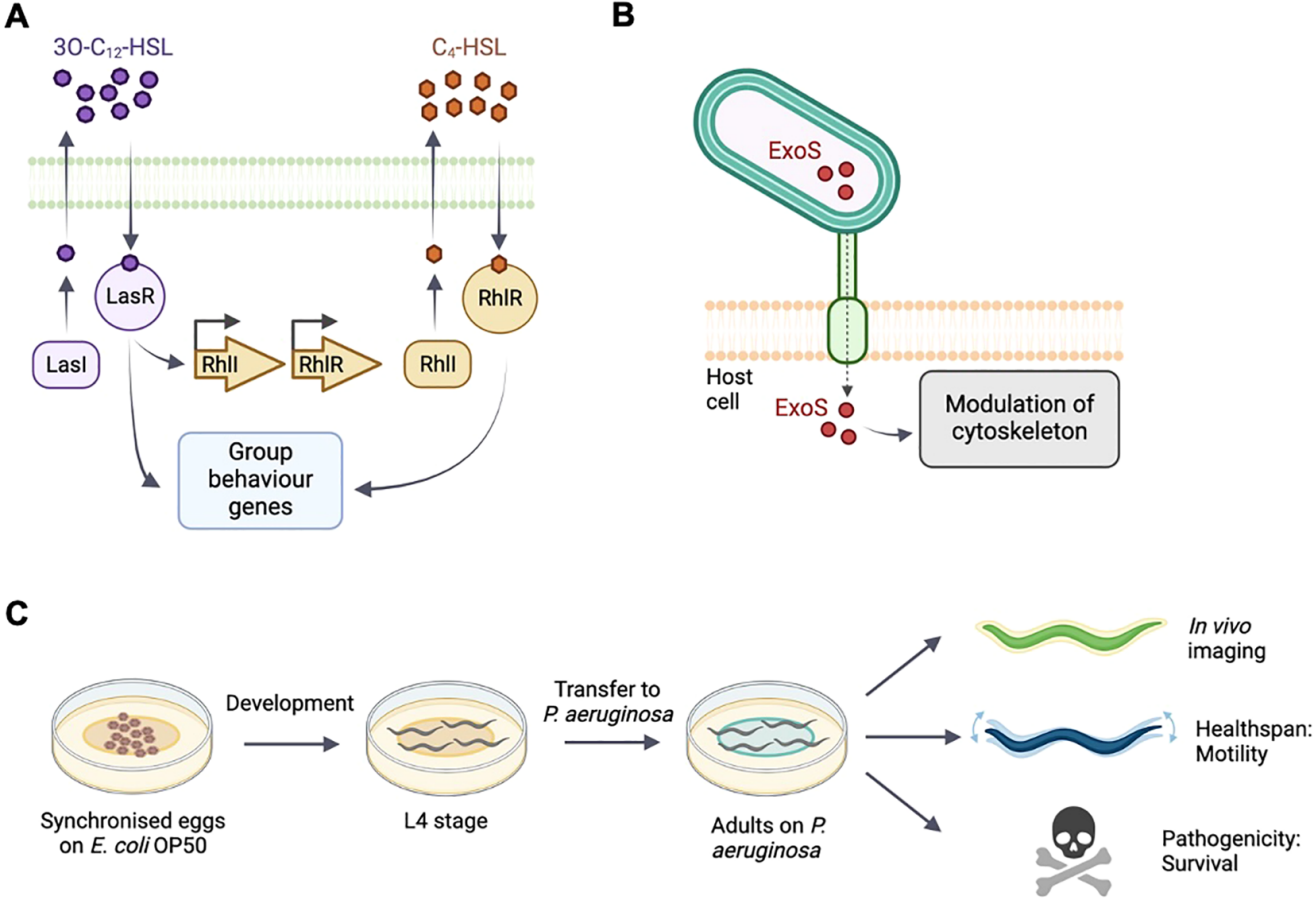
Schematic of quorum sensing and type-III secretion in *P. aeruginosa* and *C. elegans* experimental setup. **(A)** The acyl-homoserine lactone-based quorum sensing network. LasI produces and LasR responds to the autoinducer 3OC12-HSL. The LasR:3OC12–HSL complex activates transcription of many genes including rhlR. RhlI generates the autoinducer C4-HSL. **(B)** *P. aeruginosa* injects exotoxin ExoS though a needle complex after contact with the surface of the targeted eukaryotic cell. ExoS modulates the cytoskeleton. **(C)** *C*. *elegans* populations are synchronised and cultivated on *E*. *coli* OP50. At L4 stage, animals are transferred onto the different experimental bacterial strains. Bacterial fluorescence, healthspan and survival are monitored throughout adulthood.

QS enables bacteria to respond to changes in the density and composition of the surrounding bacterial community and synchronize behavioral responses through extracellular molecules, autoinducers, to produce biofilms (Thi et al., 2020). In *P. aeruginosa*, QS is regulated through the production and secretion of the autoinducers *N*-3-oxo-dodecanoyl-ʟ-homoserine lactone (3O-C_12_-HSL) and *N*-butyryl-ʟ-homoserine lactone (C_4_-HSL), which are produced by the canonical acylated homoserine lactone synthases LasI and RhlI, respectively. 3O-C_12_-HSL is sensed by the transcriptional regulator LasR and C_4_-HSL is sensed by the transcriptional regulator RhlR. Binding of the autoinducers to LasR and RhlR results in transcriptional responses and expression of genes important for biofilm formation and virulence (Mukherjee et al., 2017; O’Loughlin et al., 2013; Thi et al., 2020) (**Figure 1A**).

In this study, we use fluorescent reporters for the RhlI/R QS system to examine and monitor infection, and the resulting pathogenicity *in vivo* in *C. elegans*. We also examine a reporter of the virulence factor exoenzyme S (ExoS), a type III secretion effector which is injected into the host cell during infection, where it reorganizes the cytoskeleton and induces apoptosis (**Figure 1B**). The type III secretion system is important for acute phases of infection but a role in biofilm formation has not been clearly established (Jouault et al., 2022).

Using fluorescent *P. aeruginosa* reporters, we confirm that QS is required for *P. aeruginosa* to form biofilms *in vitro*. We combine *in vivo* epifluorescence microscopy and functional readouts using automated tracking systems (**Figure 1C**) to demonstrate that RhlR signaling is required for *P. aeruginosa* to invade *C. elegans* tissues, leading to a reduction in healthspan and survival. Our findings suggest that *P. aeruginosa* infects *C. elegans* through QS and biofilm formation, and that methods combining *C. elegans*, functional assays and bacterial fluorescent reporters can be developed into high-throughput approaches to investigate pathogenic biofilms and evaluate anti-biofilms strategies *in vivo*.

## 2 Materials and methods

### 2.1 Bacterial strains and growth conditions

Bacterial strains used in this study are listed in **Table 1**. Bacterial strains were grown in Luria-Bertani broth (LB) and on LB plates fortified with 1.5% Bacto agar at 37°C. Single bacterial colonies were inoculated and incubated at 37°C for 16 hours at 250rpm. When appropriate, antimicrobials were included at the following concentrations: 200 μg/mL carbenicillin, 25 μg/mL gentamycin, 50 μg/mL tetracycline. For assays using 4-Nitropyridine-N-oxide (NPO), the compound was dissolved in DMSO and added to bacterial cultures, keeping DMSO at a maximum of 1% (v/v).

**Table 1:** Bacterial strains used in study.

Parental strain	Strain and genotype	Additional information	Reference
*E. coli*	BL21	Control *in vitro* assays	(Daegelen et al., 2009)
*E. coli*	OP50	Control *C. elegans* assays	(Brenner, 1974)
PA103	Wildtype		
PA103	*PexoS-GFP*	Plasmid pJNE05 Gentamycin resistant	(Le Berre et al., 2008)
PA14	Wildtype		
PA14	*PexoS-GFP*	Plasmid pJNE05 Gentamycin resistant	(Le Berre et al., 2008)
PA14	SM381 *PrhlA-mNeonGreen*		(Mukherjee et al., 2017)
PA14	SM383 *ΔRhlR PrhlA-mNeonGreen*		(Mukherjee et al., 2017)

Colour relates to the genetic background (E. coli, PAO1, PA14).

### 2.2 Crystal violet staining assay

Static biofilm formation was evaluated using the crystal violet staining method following established procedure (Campo-Pérez et al., 2023). Briefly, bacterial cultures were washed with PBS three times to remove unbound cells, resuspended in Mueller Hinton broth (M-HB) and diluted to OD600 0.2. 10 µl of each sample was mixed with 190 µl of M-HB in 96-well microplate. After 2 hours of static incubation at 37°C media and non-adhered cells were removed and replaced with fresh M-HB and further incubated for 24 hrs. Adherent cells were quantified by staining with crystal violet and measurement of A_550_. The assay was performed with three biological replicates consisting of three technical replicates for each condition.

### 2.3 Fluorescence biofilm assay

Fluorescence biofilm assays were adapted from previously described methods (Alam et al., 2023). 96-well microplates with bacterial cultures were prepared as for the Crystal Violet assay but instead of staining GFP fluorescence was measured using a microplate spectrofluorometer reader. The assay was performed with three replicates consisting of three technical replicates for each condition.

### 2.4 *C. elegans* culture and strains

*C. elegans* were maintained at 15˚C on media plates seeded with *E. coli* OP50 as previously described (Brenner, 1974). For epifluorescence imaging and survival experiments, animals were cultivated using plates with 10 mL Nematode Growth Medium. For healthspan assays, plates with 15 mL defined agar were used (Maynard et al., 2018). The *C. elegans* strains used in this study was SS104 *glp-4(bn2)*, a temperature sensitive mutant which limits germline development at 20°C or above, leading to sterility.

### 2.5 Preparation of cohorts for imaging, survival and healthspan assays

An overview of preparation of *C. elegans* cohorts is shown in **Figure 1C**. For imaging and survival assays, animals were synchronized by bleaching gravid adults and placing eggs on NGM seeded with *E. coli* OP50, and incubated at 15°C. Infection by *P. aeruginosa* was conducted as described previously (Liu et al., 2024) with the exception that assay plates were kept at RT before starting the experiment. At L4 stage animals were transferred to experimental plates seeded with *P. aeruginosa* wildtype, *P. aeruginosa* reporter strains or *E. coli* OP50 control and shifted to 25°C. For healthspan assays, animals were synchronized by egg lay and transferred to experimental plates and 24°C at L4 stage. All experimental plates were seeded with 250 μL of bacterial culture and used the following day.

### 2.6 Imaging and image analysis

Animals were imaged at 24 and 96 hours after shifting L4s to experimental plates and 25°C. At each time point, 10-15 animals were mounted onto 2.5% agar pads and anesthetized with 25 mM tetramisole. DIC and fluorescence (488nm Exc./505-575nm Em) images were acquired with a CellCam Rana 200CR camera and Leica DMR compound microscope driven by Micro-Manager Studio Version 2.0.0. Brightfield and fluorescent images were stitched together in ImageJ using the Pairwise Stitching tool. The brightfield and fluorescent images were converted into an image stack. Outlines of the animals were drawn from the start to the end of the intestine (just before terminal bulb until the anus) based on the brightfield image using the Polygon Selection tool. This region was then measured in the corresponding fluorescent channel for area (pixels) and brightness (mean brightness). Size was quantified by measuring the area from neck to tail. Two biological replicates with 10-15 animals in each were performed.

### 2.7 Killing assays

Animals were shifted to experimental plates and 25°C at L4 stage and scored dead or alive at 48, 96, and 192 h (Liu et al., 2024; Mylonakis et al., 2002). Animals were considered dead if not responsive to prodding with a platinum wire. Three replicates were performed with 30 worms per replicate. Animals with internal hatching or that went missing from the plates were censored.

### 2.8 *C. elegans* healthspan assays

Healthspan was assessed using the WormGazer™ automated imaging technology (Magnitude Biosciences) (Zavagno et al., 2024). 60 mm plates were imaged using Raspberry Pi Version 2 cameras at a distance of 60 mm from the plate using white transmission illumination from a generic LED light panel. The cameras were located inside a temperature controlled laboratory set to 24°C. For each dish, a sequence of 200 images were taken over a 160 secs, with recording performed every 5 minutes until day 10 of adulthood. From these images, the number of moving objects is calculated by applying a threshold of the minimum speed of each object of 10 µm s^−1^. The speed is derived from the length of the object divided by the 160-s time interval of the imaging window. Plates were censored if they failed a quality control inspection after the experimental runtime, for example if they were contaminated with another microbe or the worms had burrowed into the agar. Censored plates were omitted from data analysis. Animals were imaged continuously using a minimum of 6 plates per condition.

### 2.9 Transmission electron microscopy

Samples were prepared as previously described (Tataridas-Pallas et al., 2021). Briefly, *C. elegans* were first fixed using 3.2% formaldehyde, 0.2% glutaraldehyde in 100mM sodium cacodylate buffer (CAB; pH 7.2). Bodies were separated from heads and tails using a scalpel and left overnight in 2.5% glutaraldehyde at 4 °C. Fixative was removed by washing, followed by re-suspension in 2% low melting-point agarose in CAB. Bodies were excised, transferred to glass vials and they were stained with 1% osmium tetroxide in CAB for 1 h at room temperature. After removal of excess stain by washing bodies were dehydrated in an ethanol series (50%, 70%, 90%, and 100%). Dehydration was followed by t washing in propelyne oxide, after which the samples were treated with a 1:1 mixture of low-viscosity (LV) resin and propelyne oxide for 30 minutes at room temperature. Samples were incubated in fresh LV resin twice for 2 hours each before being embedded in LV resin by polymerisation at 60 °C for 24 h. Embedded samples were sectioned to generate 70 nm-thick transverse sections, approximately equidistant from the vulva and the anterior tip of the worm body, using a Diatome diamond knife and an EM UC7 ultramicrotome. Sections were collected onto 400-mesh copper grids (Agar Scientific) and counterstained with 4.5% uranyl acetate for 45 minutes, followed by Reynolds’ lead citrate for 7 minutes. Sections were imaged using JEOL F200 STEM at 200 kV.

### 2.10 Statistical analysis

For healthspan measures, the difference between the means were analyzed using Gaussian error statistics and setting significance thresholds with reference to the difference expressed in terms of the standard error. Non significance is defined as a difference of less than 1.64 standard errors and significance defined as a difference of 1.64 or more. A difference between 1.64 and 2.33 standard errors is defined as one star (*), corresponding to *p* < 0.05 on a one-sided test. A difference between 2.33 and 2.83 standard errors is defines as two stars (**), corresponding to *p* < 0.01 on a one-sided test. A difference greater than 2.83 standard errors is defined as three stars (***) corresponding to *p* < 0.002 on a one-sided test. Crystal Violet and fluorescence measurements were analyzed using One-way ANOVA, and survival was analyzed using Log-rank (Mantel-Cox) tests using GraphPad Prism 10.2.3.

## 3 Results

### 3.1 *P. aeruginosa* biofilm formation *in vitro* is dependent on QS systems

We assessed the biofilm forming capacity of bacterial fluorescent reporters of the RhlI/R QS system and the virulence factor ExoS (**Table 1**). For RhlI/R we used a fluorescent transcriptional RhlA reporter fusion (*PrhlA-mNeonGreen*) in the PA14 background that targets expression during biofilm formation and infection. We also used the same reporter carrying a deletion in RhlR (*ΔrhlR PrhlA-mNeonGreen)*. The Δ*rhlR* mutant has been reported to have biofilm abnormal morphology phenotypes and attenuated virulence (Mukherjee et al., 2017). For ExoS, we used a transcriptional reporter fusion to the *exoS* promoter (*PexoS-gfp*) in PA14 and PA103 backgrounds. As controls we used *E. coli* BL21, which does not form biofilms (Wang et al., 2020) and the PA14 and PA103 wildtype strains. PA14 is a highly virulent clinical isolate and PA103 is a QS defective LasR mutant strain producing defective biofilms (Gambello and Iglewski, 1991; Haley et al., 2012; Le Berre et al., 2008).

**Figure 2:**
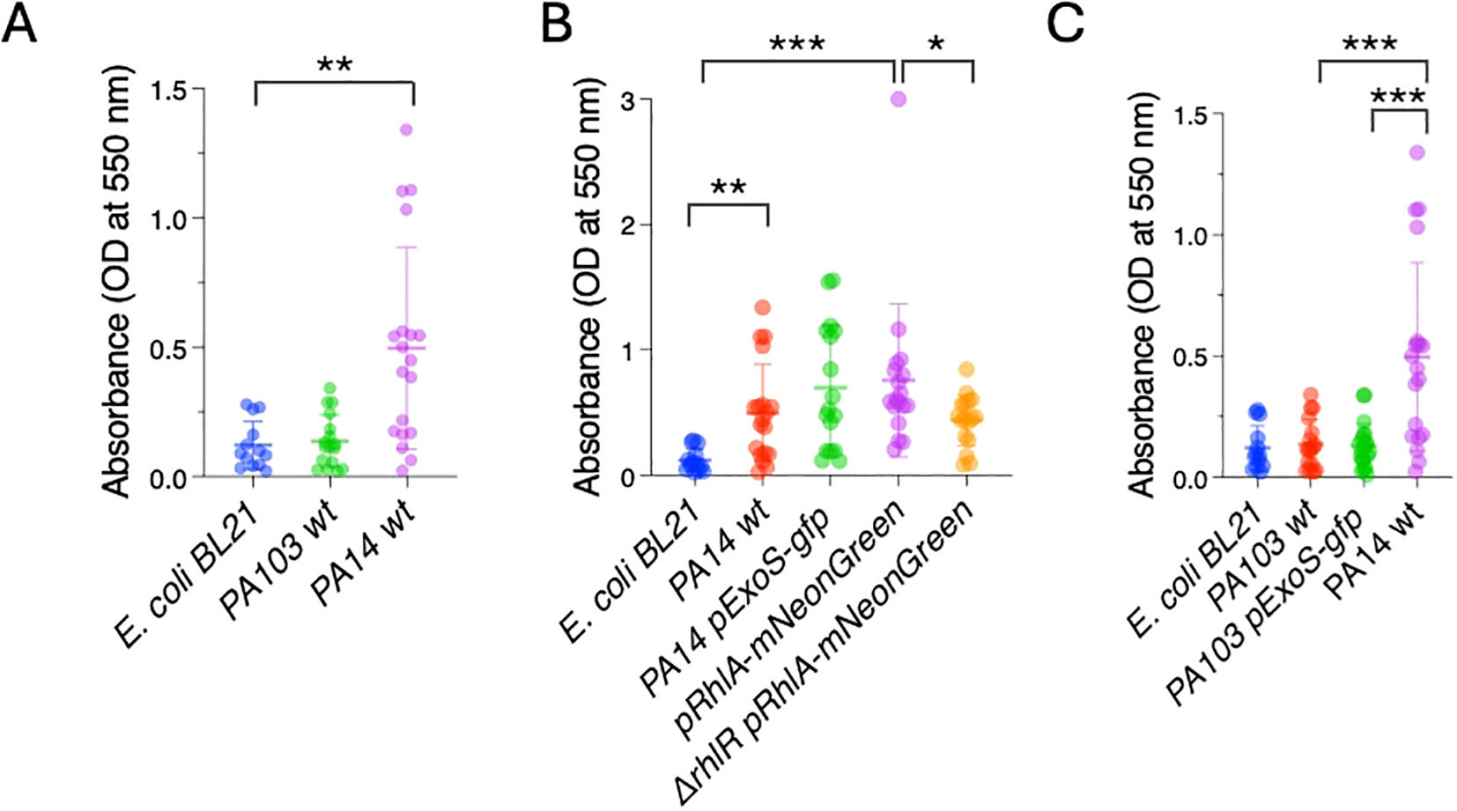
P*. aeruginosa* PA14 forms biofilms *in vitro*. Crystal violet staining was measured by absorbance at OD 550 nm in microtiter plates to quantify biofilm formation. **(A)** PA14 has high biofilm-forming capacity compared to *E*. *coli* BL21. **(B)** Reduced biofilm-forming capacity in *ΔrhlR PrhlA-NeonGreen*. **(C)** Low biofilm-forming capacity in PA103 and PA103 *PexoS-gfp*. Three biological replicates consisting of three technical replicates each were performed. Error bars represent SD of three replicates. One-way ANOVA, *p <0.05, **p <0.001, ***p <0.0001.

We first tested biofilm formation *in vitro* using the peptidoglycan stain Crystal Violet, a classical staining method used to measure biofilms (Campo-Pérez et al., 2023). The bacterial strains were inoculated in microtiter plates. After 24 hours, biofilm formation was evaluated by removing non-adherent bacteria, staining the adherent cells using Crystal Violet, and measuring absorbance at 550 nm. PA14 showed a 4.2-fold increase in absorbance in comparison to the biofilm incompetent *E. coli* BL21, in agreement with good biofilm forming ability. In contrast, PA103 had similar staining levels as *E. coli* BL21, confirming a low biofilm forming capacity (**Figure 2A**). The *PexoS-gfp* and *PrhlA-NeonGreen* reporters in PA14 background showed absorbance levels similar to PA14 wildtype. *ΔrhlR PrhlA-NeonGreen* had a 1.7-fold lower absorbance compared to *PrhlA-NeonGreen*, and PA103 and PA103 *PexoS-gfp* had absorbance levels similar to *E. coli* BL21, suggesting compromised biofilm-forming abilities in these strains (**Figures 2B, C**). These findings confirm the biofilm forming capacity of PA14 and central role of the RhlI/R quorum sensing systems for the formation of *P. aeruginosa* biofilms.

### 3.2 *P. aeruginosa* reporters form fluorescent biofilms *in vitro*

**Figure 3:**
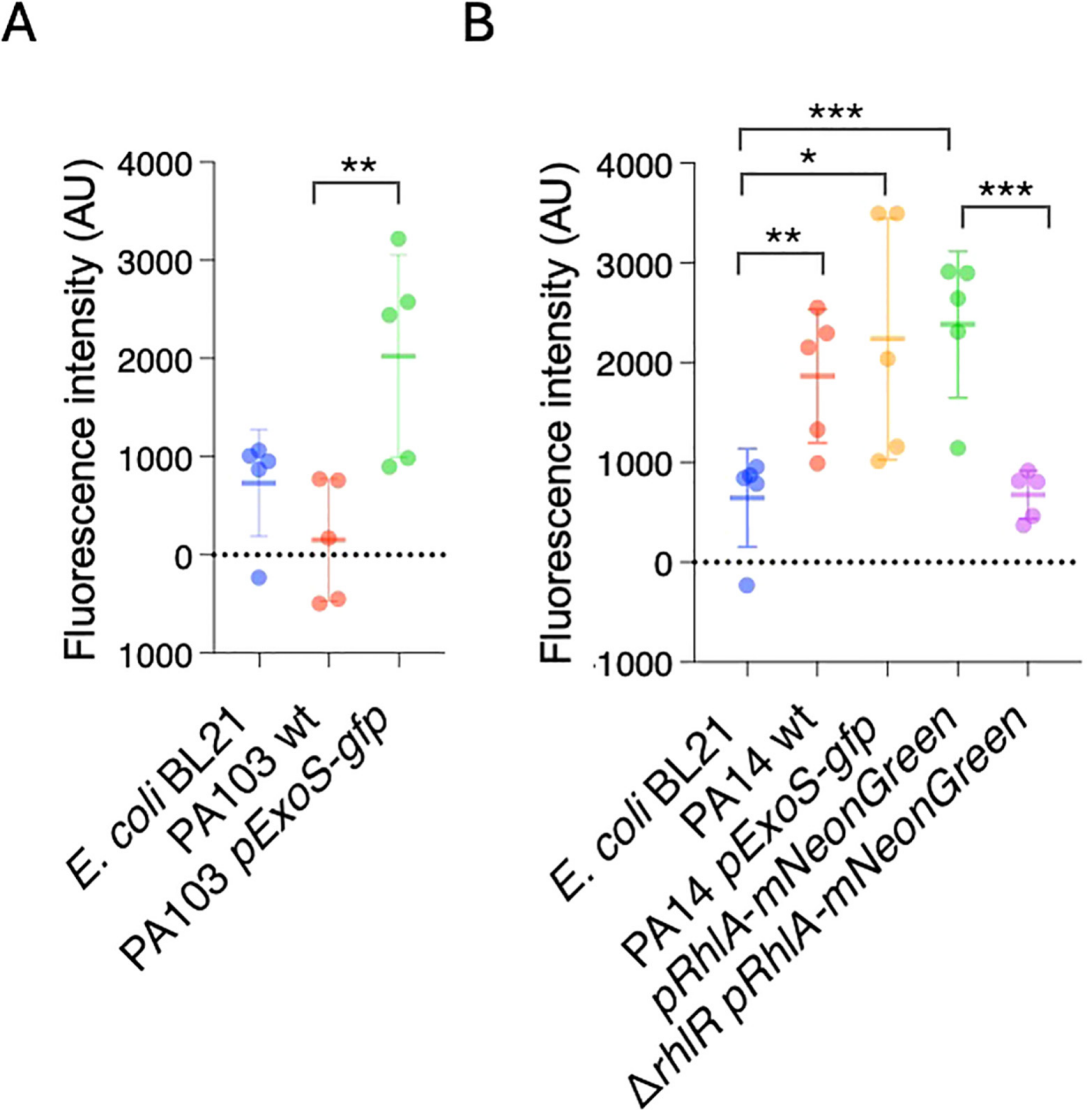
P*. aeruginosa* reporters form fluorescent biofilms *in vitro.* GFP fluorescence was measured in microtiter plates to quantify biofilm formation. **(A)** Increased fluorescence in PA103 *PexoS-gfp* compared to PA103. **(B)** Fluorescence in *PrhlA-NeonGreen* reduced by *rhlR* mutation. Three biological replicates consisting of three technical replicates each were performed. Error bars represent SD of three replicates. One-way ANOVA, *p <0.05, **p <0.001, ***p <0.0001.

Next, we tested if the biofilms can also be detected by measuring fluorescence produced by the reporters. Bacterial strains were inoculated in microtiter plates, and after 24 hours planktonic cells were washed off. Biofilms were quantified by measuring fluorescence at 488 nm/507 nm. *E. coli* BL21 and PA103 had low levels fluorescence relative to PA103 *PexoS-gfp*, which showed a 13.14-fold increase in fluorescence compared to PA103 background (**Figure 3C**). PA14 had significantly higher levels of fluorescence compared to *E. coli* BL21 (**Figure 3B**), likely due to autofluorescence resulting from pyocyanin production (Mukherjee et al., 2017). PA14 *PexoS-gfp* and *PrhlA-NeonGreen* fluorescence was 3.1 and 3.7-fold higher than *E. coli* BL21, respectively, but not significantly different from PA14 wildtype. *PrhlA-NeonGreen* fluorescence was reduced by 3.5-fold in the *ΔrhlR* mutant (**Figure 3B**).

Except for PA103 *PexoS-gfp*, the reporters yielded similar results across both biofilm assays. The PA103 *PexoS-gfp* showed high levels of fluorescence compared to PA103, but similar levels of absorbance in the Crystal Violet assay, indicating discrepancies between the two methods for this strain. The fluorescence assay was not able to detect differences in between reporters and PA14 wildtype, likely due to overlapping spectra between the reporters and background fluorescence arising from autofluorescence.

### 3.3 Infection by PA14 can be visualized using of ExoS and RhlA reporters

After measuring biofilms produced by the reporters *in vitro*, we asked if the reporters could be used to visualize bacterial infection *in vivo* in *C. elegans*. *C. elegans* is transparent, allowing imaging living animals without killing or dissection, making it ideal to monitor infection using fluorescent reporters. We cultivated *C. elegans* with the laboratory standard, non-pathogenic bacteria *E. coli* OP50 during development and exposed animals to the *P. aeruginosa* strains at L4 stage (end of development). Fluorescence was monitored *in vivo* using epifluorescence imaging 24 and 96 hours after exposure to *P. aeruginosa*. The later timepoint was chosen to capture early phases infection. We also attempted to image 6 days after exposure but found that these timepoints were not suitable, since for some of the conditions the surviving animals were very fragile, leading to bursting and death when mounted on slides.

**Figure 4:**
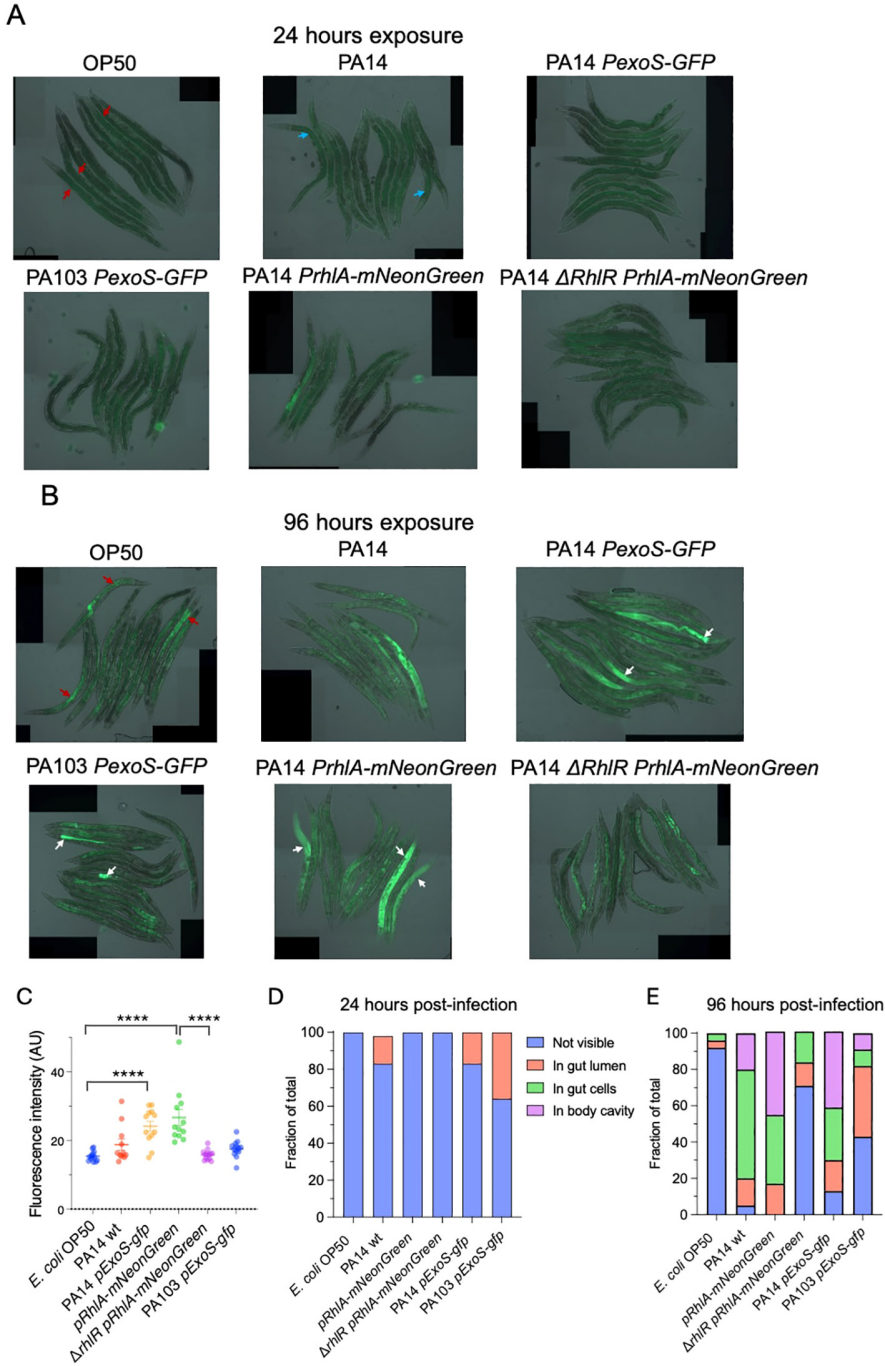
*In vivo* imaging of *C*. *elegans* exposed to *P. aeruginosa* reporter strains. **(A)** Overlay of DIC and epifluorescence images taken after 24 hours of exposure. Autofluorescence from C. elegans gut granules within the intestinal cells (red arrows). Autofluorescence from *P. aeruginosa* pyocyanin within the intestinal lumen (blue arrows). **(B)** Overlay of DIC and epifluorescence images taken after 96 hours of exposure. Widespread fluorescence observed in animals exposed to PA14 *PexoS-gfp* and *pRhlA-NeonGreen* (white arrows). Widespread fluorescence absent in *ΔrhlR* mutant background. PA103 *PexoS-gfp* fluorescence is contained within the intestinal lumen (white arrows). Autofluorescence from *C*. *elegans* gut granules within the intestinal cells (red arrows). **(C)** Quantification of mean fluorescence intensity. Two-way ANOVA was used for statistical comparisons. **(D, E)** Fraction of animals where bacterial fluorescence was not visible, visible in gut lumen, visible in gut cells or visible in body cavity at **(D)** 24 hours and **(E)** 96 hours post-infection. Fischer exact probability test was used for statistical comparisons (results described in main text). Two biological trials with 10-15 animals in each were conducted. ****p <0.0001.

In *C. elegans* the primary entry point for bacteria and site of infection is the intestine via the upper part of the gastro-intestinal tract, which consists of the mouth and pharynx. The intestine contains lysosome-related granules that generate autofluorescence. We observed fluorescence within the gut granules in all experimental conditions. In animals exposed to *E. coli* OP50 fluorescence was not seen in the intestinal lumen nor in other tissues. At 24 hours after exposure, there were only minor differences in fluorescence between the experimental conditions (**Figures 4A, D**). In contrast, after 96 hours different patterns of fluorescence could be observed. In animals infected with PA14 *PexoS-gfp*, fluorescence could be seen in the intestinal lumen, consistent with it coming from GFP derived from bacteria proliferating in the lumen. In some animals’ widespread fluorescence, not only localized to gut granules, was present in the intestine as well as in the body cavity, indicating GFP-expressing bacteria had crossed the intestinal barrier and entered other tissues. Animals exposed to *PexoS-gfp* in PA103 background showed fluorescence contained to gut granules, with a subset of animals also having fluorescence present in the intestinal lumen. No fluorescence was observed in the body cavity, suggesting a reduced ability of PA103 to translocate through the intestine and infect the animal compared to PA14. Animals exposed to PA14 *pRhlA-NeonGreen* had fluorescence present in the intestinal lumen, intestinal cells and body cavity, while in inanimals exposed to *ΔrhlR* PA14 *pRhlA-NeonGreen* fluorescence was not observed in the body cavity (**Figures 4B, E**).

Quantification of mean fluorescence at 96 hours post-infection showed that fluorescence intensity was increased in animals infected with PA14 and PA103 strains compared to OP50 controls. The *ΔrhlR* mutation significantly decreased mean intensity compared to PA14 *pRhlA-NeonGreen* and significantly altered the distribution of fluorescence between PA14 *pRhlA-NeonGreen* and *ΔrhlR* PA14 *pRhlA-NeonGreen* (**Figures 4C, E**). Distribution was also significantly different when comparing OP50 to PA14 wt, PA14 *PexoS-gfp* and PA103 *PexoS-gfp* but not compared to *ΔrhlR* PA14 *pRhlA-NeonGreen*, and significantly different between PA14 *PexoS-gfp* and PA103 *PexoS-gfp* (**Figure 4E**). Thus we could observe differences both in distribution and intensity of the fluorescent reporters *in vivo*.

Together our imaging data show that by 96 hours of exposure, PA14 can cross the intestinal epithelial barrier to invade tissues, while for PA103 background the bacteria remain within the lumen. Translocation by PA14 can be visualized using the *PexoS-gfp* and *pRhlA-NeonGreen* reporters and is dependent on *RhlR*, confirming quorum sensing is important for PA14 virulence in *C. elegans*.

### 3.4 Reduction of RhlR signaling decreases PA14 pathogenic effects on lifespan and healthspan

**Figure 5:**
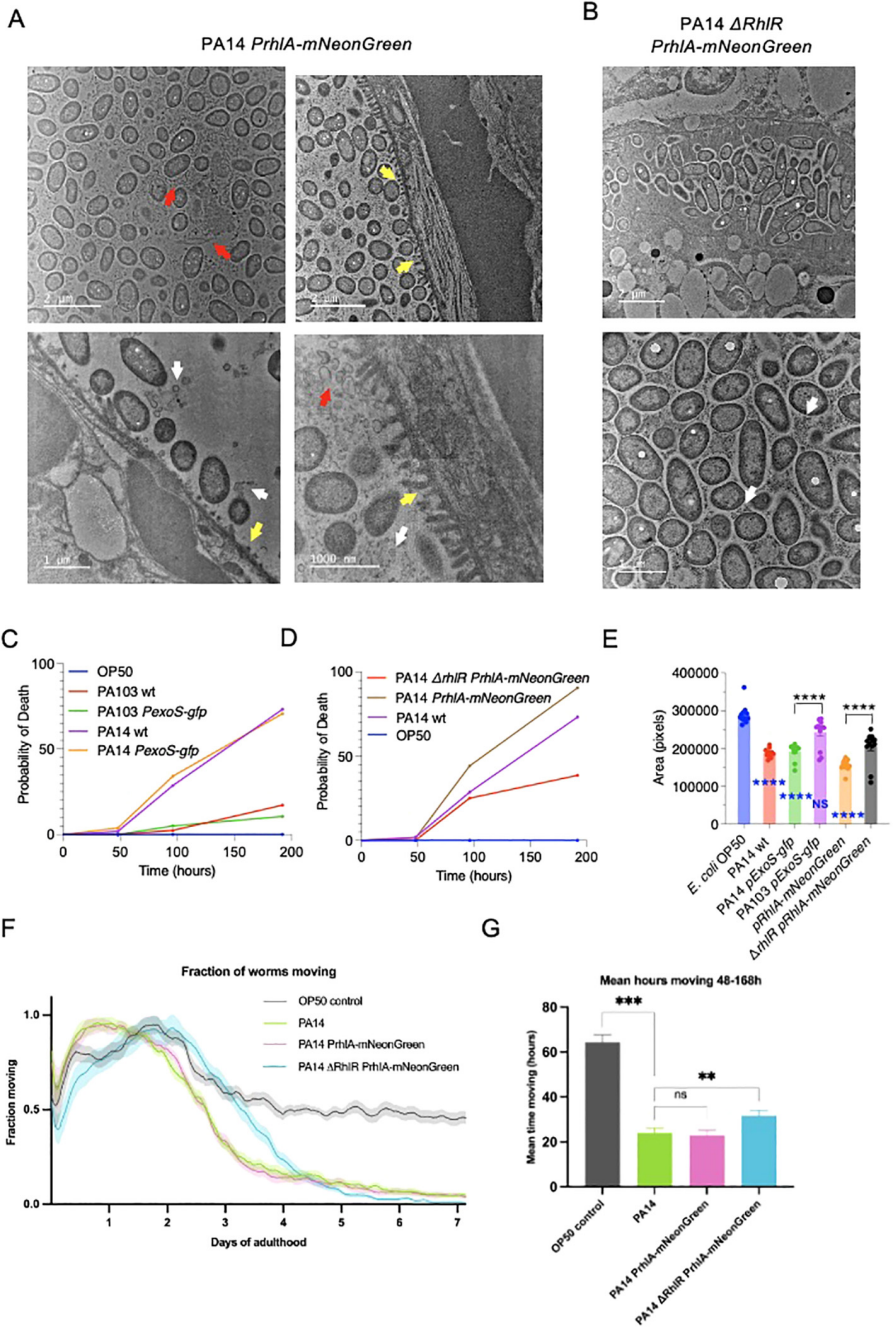
Decreased *RhlR* signalling in *P. aeruginosa* reduces pathogenicity in *C. elegans.* **(A, B)** Representative cross-sectional transmission electron micrographs of *C. elegans* infected with PA14 *pRhlA-NeonGreen* and *ΔrhlR pRhlA-NeonGreen* **(A)** Degraded and broken microvilli (yellow arrows), bacterial vesicles (white arrows) and extracellular material (red arrows) visible in PA14 *pRhlA-NeonGreen* infected animals. **(B)** In PA14 *ΔrhlR pRhlA-NeonGreen* infected animals broken microvilli are not visible. Some vesicle observed (white arrows). Extracellular material is present but appears structurally different. Bacterial cells are more densely packed and are penetrating the intestinal brush border. N=2 per condition. **(C, D)** *C. elegans* survival was monitored up to 192 hours from L4 stage. Exposure to PA14 strains resulted in decreased survival, which was reduced by *ΔrhlR*. Exposure to PA103 did not affect survival. Three biological replicates with n=30 were performed. Log-rank (Mantel-Cox) test was performed for comparison of survival. **(E)** Measurement of *C. elegans* body area after 96 hours of exposure to bacteria. Two biological trials with 10-15 animals in each were conducted. Two-way ANOVA. Blue indicates comparison with OP50. *p <0.05, *p <0.01, **p <0.001, ****p <0.0001. **(F, G)** *C. elegans* healthspan assays were performed by monitoring motility for 7 days starting at L4 stage. Exposure PA14 and PA14 *PrhlA-mNeonGreen* reduced motility compared to *E. coli* OP50. Animals exposed with to *ΔrhlR PrhlA-mNeonGreen* remained mobile for longer. **(F)** Fraction of animals moving in healthspan assay. Shading shows SEM. **(G)** The area under the curve integration for hours 48-168 of the healthspan assay. Error bars show SEM. Two biological replicates with n=180 were performed. **p<0.01, ***p<0.002, one-tailed t-test.

To further visualize how host-pathogen interactions are affected by *RhlR* we used transmission electron microscopy (TEM). *C. elegans* were fixed after 96 hours of exposure to *E. coli* OP50, PA14 *pRhlA-NeonGreen* or *ΔrhlR* PA14 *pRhlA-NeonGreen*. In animals exposed to *E. coli* OP50, only very few bacterial cells were observed, consistent with OP50 not being pathogenic, and cells being digested by the host. In contrast, in animals exposed to both PA14 strains bacterial cells were numerous, distending the gut (**Figures 5A, B**). Noticeably, in animals exposed to PA14 *pRhlA-NeonGreen*, intestinal microvilli were degraded, shortened and broken, while animals exposed to PA14 *ΔrhlR* had intact microvilli. PA14 *pRhlA-NeonGreen* cells appeared less densely packed compared to PA14 *ΔrhlR* mNeongreen, suggesting the presence of an extracellular matrix separating the cells and consistent reduced biofilm-forming capacity resulting from *ΔrhlR*. In addition, we observed considerable amounts of extracellular material between the bacterial PA14 *pRhlA-NeonGreen* cells within the *C. elegans* gut, e.g. membrane vesicles and cellular debris. The extracellular material between PA14 *ΔrhlR pRhlA-NeonGreen* appeared qualitatively distinct and with fewer vesicles present (**Figure 5B**), consistent with the abnormal biofilm morphology phenotypes reported for *ΔrhlR* mutants (Mukherjee et al., 2017). Although degraded microvilli were not observed in PA14 *ΔrhlR pRhlA-NeonGreen* infected animals, bacterial cells were in direct contract to the microvilli surface, appearing to be in the process of penetrating the intestinal wall (**Figure 5A**).

The differences we observed between the reporters in biofilm-formation capacity, ability to cross the intestinal barrier and bacteria-host interactions, prompted us to examine virulence resulting from infection with the strains. We first conducted killing assays to determine if strains that translocate into the body cavity are more lethal than those that do not. Animals were cultivated on *E. coli* OP50 and transferred to *P. aeruginosa* strains at L4 stage and survival was monitored at 48, 96 and 192 hours (8 days). Survival of control animals kept on *E. coli* OP50 was not affected during the experiment, and exposure to PA103 and PA103 *PexoS-gfp* resulted in a small, non-significant reduction in survival. In contrast, infection with wildtype PA14 and PA14 *PexoS-gfp* resulted in 73.2% and 71.6% of animals dead at 192 hours (**Figure 5C**). Exposure to PA14 *pRhlA-NeonGreen* resulted in 91.6% of animals dead while *ΔrhlR* reduced lethality of PA14 *PrhlA-mNeonGreen*, animals exposed to this strain had a survival of 61.4% at 196 hours (**Figure 5D**). These data are consistent with observations in our imaging experiments; PA103 is does not invade *C. elegans* tissues and has limited pathogenicity, while PA14 is capable of crossing the intestinal barrier and is highly pathogenic. PA14 pathogenicity is dependent on *RhlR*, consistent with RhlR signaling being important for virulence. Pathogenicity of PA14 *PexoS-gfp* and PA14 *PrhlA-mNeonGreen* were not different from PA14, thus expression of the reporters does not affect PA14 virulence.

*C. elegans* infection models typically test the ability of pathogens to kill the host, while effects on overall physical fitness are usually not assessed. We asked if PA14 pathogenicity in addition to affecting survival, also impacts on fitness. We first compared body size in animals exposed to OP50 and PA14, and found that PA14 reduces body area by 34.6% (**Figure 5E**), suggesting effects on host fitness. Comparison of the reporter strains showed that PA14 *pExoS-gfp* and PA103 *pExoS-gfp* reduced body size by 31.7% and 11.7% respectively compared to OP50. Body area in animals exposed to PA14 *pExoS-gfp* and PA103 *pExoS-gfp* were significantly different from each other (22.6%), and compared to *ΔrhlR pRhlA-mNeonGreen*, *pRhlA-mNeonGreen* reduced body size by 25.7% (**Figure 5E**). The changes in body size mirror the effects we observed in survival and are consistent with PA14 biofilms affecting host fitness.

Next we used automated tracking and image analysis (Zavagno et al., 2024) to assess healthspan. We profiled motility of animals following infection with *P. aeruginosa*, comparing PA14 wildtype, PA14 *PrhlA-mNeonGreen* and PA14 *ΔrhlR PrhlA-mNeonGreen*. Animals were tracked continuously from L4 stage until day 7 of adulthood to measure fraction animals moving, and hours spent moving and compared to *E. coli* OP50 controls. Animals in all conditions were active and mobile during the first two days of tracking, as expected in young, healthy animals (**Figure 5F**). Animals grown on OP50 showed a partial reduction in movement between days 2 and 4 of adulthood due to normal age-related decline in muscle function (Huang et al., 2004; Newell Stamper et al., 2018). PA14 and PA14 *PrhlA-mNeonGreen* reduced overall movement and the time spent moving (**Figures 5F, G**). Animals exposed to PA14 *ΔrhlR PrhlA-mNeonGreen* exhibited a prolonged healthspan compared to PA14 and PA14 *PrhlA-mNeonGreen*, increasing both the distance moved and time spent moving (**Figures 5F, G**). Thus *P. aeruginosa* negatively affects both survival and healthspan and these effects are reduced when QS is compromised.

## 4 Discussion

*P. aeruginosa* biofilms have been studied extensively on glass or plastic surfaces *in vitro*, but much less is known about how biofilms form and develop on an epithelial barrier, the most common site of infection. In our study we infected the genetically tractable and transparent model organism *C. elegans* with fluorescent *P. aeruginosa* reporters to monitor infection *in vivo* while providing readouts of host health. By combining *in vivo* microscopy methods with new automated tracking technology, we show that the transcriptional RhlR receptor is important for the ability of *P. aeruginosa* to invade host tissues, and for pathogenic effects on survival and healthspan. As *P. aeruginosa* uses the same invasion mechanism across different host species, and mammals and invertebrates use conserved signal transduction pathways to activate defense-related genes (Tan et al., 1999), *C. elegans* is able to model certain aspects of mammalian pathogenesis. Our study is consistent with other studies examining *P. aeruginosa* infections and showing that RhlR plays central role in biofilm formation and virulence (Mukherjee et al., 2018; Kumar et al., 2021). For example, mutations in the RhlR transcription factor receptor alter biofilm morphology and reduce the activation of host anti-pathogen defenses (Peterson et al., 2023). In another study, comparing wildtype *P*. *aeruginosa* with an *ΔrhIR* mutant, mice were exposed to an intratracheal challenge and bacterial colonization was monitored in real time. The study showed that the Δ*rhIR* mutation attenuates virulence and bacterial load while also reducing pathogenicity in *C. elegans* (Mukherjee et al., 2017). Thus, there are parallels between findings in the murine lung infection and *C. elegans* models and using *C. elegans* as pre-clinical model has the potential to accelerate the development of new antimicrobial strategies.

Click or tap here to enter text. Click or tap here to enter text.

*In vivo* imaging infection of *C. elegans* with the *PrhlA-mNeonGreen* reporters showed that the widespread infection in wildtype background was absent in the animals exposed to the mutant, consistent with the compromised biofilm forming capacity in *rhlR* mutants resulting in reduced virulence. Using standard biofilm assays, we confirmed that the *rhlR* deletion reduces *P. aeruginosa* biofilms *in vitro*. *In vitro* the autofluorescence generated by pyocyanin production meant there were no differences in overall fluorescence levels between PA14 and the *PrhlA-mNeonGreen* reporter. In contrast, we could observe clear differences in pattern and distribution of bacterial fluorescence *in vivo*.

Also, the *PexoS-gfp* reporter in PA14 background showed a high degree of biofilm formation capacity combined with pathogenicity. As with the *mNeonGreen* reporter, we could not detect differences in fluorescence intensity between wildtype PA14 and PA14 *PexoS-gfp in vitro* due to autofluorescence, but found major differences in bacterial fluorescence *in vivo*, with widespread tissue fluorescence following PA14 *PexoS-gfp* infection. This fluorescence was not observed in animals exposed to PA103 *PexoS-gfp*, and PA103 was also less pathogenic. In mice models of acute pneumonia, PA103 is highly virulent, severely reducing survival rates within 24 hours of exposure (MaChado et al., 2010). The moderate pathogenicity of PA103 and lack of intestinal infection in our *C. elegans* assays highlight species-specific differences and raise the question of why *C. elegans* is able to avoid infection. A study using two different models of infection in *Drosophila* showed that PA103 results in high lethality in a fly nicking model but not in a feeding model (L. et al., 2008). As PA103 lacks a functional QS system, QS might be required for lethality by feeding, e.g. to successfully attach to and infect intestinal cells. This can explain the lack of virulence in our *C. elegans* experiments were we also used feeding as means to expose the animals to the bacteria.

*P. aeruginosa* is listed as a high-priority pathogen in the 2024 WHO Bacterial Priority Pathogens List, due to its growing antibiotic resistance and global threat, especially in health-care settings (WHO Bacterial Priority Pathogens List, 2024). Currently very few treatments targeting *P. aeruginosa* biofilms are in development (Reynolds and Kollef, 2021) and the need for developing effective treatments against antibiotic resistant pathogens calls for *in vivo* models that are rapid and can be used in high throughput. Our work provides insight into how *C. elegans* combined with low-cost epifluorescence *in vivo* imaging and automated tracking technologies can be used to study *P. aeruginosa* infection. We envision the approaches described here being developed into high-throughput *in vivo* methods using e.g. 96-well plates and compound libraries, to screen for novel antimicrobials targeting *P. aeruginosa* biofilms, improving efficiency of pre-clinical antimicrobial testing and reduce costs, timelines and ethical burdens.

Future studies could make use of fluorescent reporters with emission wavelengths that do not overlap with those of pyocyanin and gut granules to more clearly differentiate between expression of the reporter and background. It would also be useful to develop methods to assess bacterial viability in the *C. elegans* gut and body cavity, such as BacLight, and to confirm the presence of biofilms, e.g. biofilm staining methods. Overall, our work suggests that using *C. elegans* to study QS and biofilms could provide an exciting path forward for the development of novel antimicrobials.

## Data Availability

The raw data supporting the conclusions of this article will be made available by the authors, without undue reservation.
